# How Symptomatic Should a Hypertrophic Obstructive Cardiomyopathy Patient Be to Consider Alcohol Septal Ablation?

**DOI:** 10.1161/JAHA.117.006292

**Published:** 2017-05-16

**Authors:** Brandon M. Jones, Amar Krishnaswamy, Nicholas G. Smedira, Milind Y. Desai, E. Murat Tuzcu, Samir R. Kapadia

**Affiliations:** ^1^ Department of Cardiovascular Medicine Cleveland Clinic Cleveland OH; ^2^ Department of Cardiothoracic Surgery Cleveland Clinic Cleveland OH; ^3^ Department of Cardiovascular Medicine Cleveland Clinic Abu Dhabi UAE

**Keywords:** Editorials, hypertrophic cardiomyopathy, septal ablation, Hypertrophy, Cardiomyopathy, Catheter-Based Coronary and Valvular Interventions, Percutaneous Coronary Intervention

## Introduction

Hypertrophic cardiomyopathy (HCM) is a complex and heterogeneous disease with different anatomical variants, physiologic manifestations, and genetic underpinnings. Even asymptomatic patients with HCM are at potential risk for sudden cardiac death and require risk stratification and consideration of an implantable cardioverter‐defibrillator for primary or secondary prophylaxis. However, the progression to symptomatic heart failure depends most often on upper septal hypertrophy with systolic anterior motion of the anterior mitral valve leaflet causing left ventricular outflow tract (LVOT) obstruction, elevated gradients, mitral regurgitation, and often atrial fibrillation. Symptoms in patients with LVOT obstruction usually manifest as exertional dyspnea or chest pain, and β‐blockers or calcium channel blockers are the mainstay of medical therapy to reduce LV contractile forces and associated LVOT gradients. When symptoms are refractory to medical management, surgical myectomy or alcohol septal ablation (ASA) is considered, and both have been shown to be effective in carefully selected patients.^1^ Nonrandomized studies have determined that patients after surgical myectomy or ASA have an expected long‐term survival that is comparable to that of the general population, and superior to similar patients who do not undergo surgery.^2^, ^3^ Historically though, these procedures have been reserved for patients with severe symptoms and LVOT gradients ≥50 mm Hg, with the primary objective of symptom improvement, as reflected in major society guidelines.^4^, ^5^

In the current study by Veselka and colleagues in this issue of *JAHA*, the authors report the retrospective outcomes of 161 patients enrolled in the Euro‐ASA registry from 1996 to 2016 who underwent ASA despite being classified as only having New York Heart Association (NYHA) Class II symptoms.^6^ All patients had LVOT obstruction with gradients ≥50 mm Hg at rest or after provocation, and none reported angina or syncope. Importantly though, all patients reported symptoms that were refractory to medical therapy and a significantly reduced quality of life because of dyspnea. Reassuringly, the authors demonstrate that ASA in this population was safe with a 0.6% mortality at 30 days and a 9.4% rate of permanent pacemaker implantation, and show that long‐term mortality was comparable to a matched cohort in the general population. Patients in the study were followed for 895 patient‐years (median 4.8 years) of follow‐up. ASA was shown to be effective, with 88% of patients experiencing sustained resting gradient reductions ≤30 mm Hg at last follow‐up with only 9.3% of patients requiring repeat septal reduction (only 4 of whom went on to surgical myectomy). Importantly, the patients' symptoms seemingly improved, with 69% reporting NYHA Class I symptoms at last follow‐up, and only 2% of patients progressing to NYHA Class III symptoms. Therefore, the authors conclude that ASA was safe and effective in treating patients with symptomatic, medically refractory HCM with significant resting obstruction and NYHA Class II symptoms in this retrospective analysis.

There are several limitations of this retrospective study that should be mentioned. The first is that the authors do not have data on the use of provocative maneuvers to measure LVOT gradients postablation. The second is that we do not have data on the use of medications before or after ASA, which is a potential confounding variable, although we would have to assume that all patients were being treated with maximally tolerated doses. Finally, clinical improvement is only reported based on NYHA Class without other, objective measurements of quality of life or exercise capacity being available.

So how should the results of the current study be interpreted and applied? First, it must be noted that septal reduction procedures, either surgical or percutaneous, have not been shown in a randomized clinical trial to reduce long‐term mortality, and there are no data that support these invasive procedures in asymptomatic patients.^4^ There are, however, data to show that LVOT obstruction increases mortality risk in patients with hypertrophic obstructive cardiomyopathy and that after myectomy, mortality is similar to that of patients without LVOT obstruction, suggesting potential mortality benefit of myectomy. However, because of this type of indirect evidence, the primary objective of any intervention is to improve symptoms or quality of life. The current study is consistent with this recommendation, as all patients had both objectively severe gradients, and symptoms that significantly impacted their quality of life. In fact, the severity of obstruction in this cohort was quite high with a mean resting LVOT gradient of 63±32 mm Hg. Thus, despite being labeled as NYHA Class II, it is perhaps somewhat unfair to consider these patients mildly symptomatic. By definition, Class II symptoms are described as a slight limitation of physical activity, comfortable at rest, but ordinary physical activity results in symptoms of heart failure.^7^ Comparatively, Class III symptoms are defined as marked limitation of physical activity, comfortable at rest, but less than ordinary activity causes symptoms of heart failure.

There are obvious, potential limitations to using NYHA Class alone in evaluating patients with heart failure. Self‐reported symptoms are inherently subjective, and it is well known that some patients under‐report the severity of their limitations, especially when a decline is experienced gradually or over a long period of time. Thus, it is no surprise that society guidelines do not place a specific requirement for NYHA Class as a criterion for considering septal reduction techniques, and the severity of symptoms is left to the clinician to determine based on all available tools. As such, there has been much interest in studying more granular or more objective tools to help further stratify and follow patients with heart failure over time (Table). Cardiopulmonary exercise testing has been an important tool in the evaluation of patients with valve disease and hypertrophic obstructive cardiomyopathy when more mild symptoms are reported. In 1 such study, peak VO_2_ had a significant association with NYHA class, but the authors noted considerable overlap between NYHA classes I through III, indicating that many patients with significantly abnormal VO_2_ were classified as NYHA Class I or II only.^8^ Other parameters have been shown to be predictive of outcomes in patients with NYHA Class I and II symptoms undergoing exercise stress testing including achieved metabolic equivalents and heart rate recovery.^9^ The 6‐Minute Walk Test is an objective measure of exercise capacity that can be used, and is especially effective in following patients over time.^10^ Other tools have been developed to better quantify the subjective effects of hypertrophic cardiomyopathy on quality of life such as the Kansas City Cardiomyopathy Questionnaire, which may be useful in this population as poor quality of life was cited as a strong factor in patients undergoing ASA in the current study.^11^, ^12^ Finally, even a simple measurement of plasma natriuretic peptide (brain natriuretic peptide) has been shown to correlate with both NYHA Class and mortality in patients with HCM, and can be another useful tool in patients with questionable symptoms.^13^

**Table 1:** Methods for Evaluating Intermediately Symptomatic Patients With Hypertrophic Cardiomyopathy and Obstruction

Method	Description
New York Heart Association (NYHA) Class	Class I: No limitation of physical activity. Class II: Slight limitation of physical activity. Comfortable at rest. Ordinary physical activity results in fatigue, palpitation, or dyspnea. Class III: Marked limitation of physical activity. Comfortable at rest. Less than ordinary activity causes fatigue, palpitation, or dyspnea. Class IV: Unable to carry on any physical activity without discomfort. Symptoms of heart failure at rest. If any physical activity is undertaken, discomfort increases
Kansas City Cardiomyopathy Questionnaires (KCCQ)	23‐item, self‐administered questionnaire. Clinical score includes physical limitations and total symptom frequency and burden. Overall Score includes the clinical score plus measures of the stability of symptoms, self‐efficacy or perceived ability to manage symptoms as an outpatient, quality of life, and social limitations. Also available as a short form
Cardiopulmonary Exercise Testing	Symptom‐limited treadmill exercise with respiratory gas analysis. Peak VO_2_ is measured over 30‐s intervals during the test and ventilatory threshold (the point where body demands exceed the capacity for aerobic metabolism) is calculated
6‐Minute Walk Test	Measures distance walked in a 6‐minute time period
NT‐Pro‐BNP	Blood test that is elevated in a variety of heart failure conditions

NT‐Pro‐BNP indicates N‐terminal pro‐brain natriuretic peptide.

Thus, in considering treatments for patients with HCM, we advocate for a comprehensive approach to symptom evaluation using both subjective and objective tools (Figure). Patients with seemingly intermediate symptoms should be evaluated by additional methods including cardiopulmonary exercise testing, 6‐Minute Walk Testing, measurement of natriuretic peptide, and/or completion of the Kansas City Cardiomyopathy Questionnaire. When these tests reveal limitations that are out of proportion to patient's self‐reported symptoms, consideration could be made to more aggressive medical management and possibly referral for septal reduction techniques. Careful tailoring of the procedural approach for such patients requires extensive expertise with these techniques, and a heart team approach involving the input of cardiac surgeons, interventional cardiologists, cardiac anesthesiologists, and cardiovascular imaging specialists is paramount to appropriate patient selection. Furthermore, the current American College of Cardiology/American Heart Association valve guidelines advocate for establishment of Heart Valve Centers of Excellence for treating complex patients, a recommendation that would certainly be relevant to the management of complex patients with HCM.^14^ In addition to demonstrating adequate procedural volume, low procedural complication rates, and high rates of procedural success, centers of excellence would have a mandate for active participation in data registry reporting and quality improvement processes, which are increasingly important as procedures become applied to lower‐risk populations. Just as utilization of a more comprehensive approach to preablative monitoring and determining candidacy for septal reduction therapies is warranted, it is important to follow patients after ASA or myectomy with similarly objective measures, including the use of standardized provocation techniques for measuring LVOT gradients, rather than relying on NYHA Class alone to demonstrate success.

**Figure 1:**
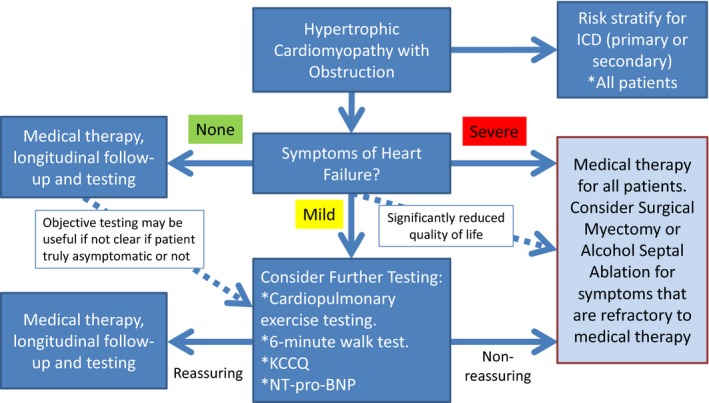
Algorithm for evaluating patients with hypertrophic cardiomyopathy with significant obstruction based on the severity of symptoms. ICD indicates implantable cardioverter defibrillator; KCCQ, Kansas City Cardiomyopathy Questionnaire; NT‐proBNP, N‐terminal pro‐brain natriuretic peptide.

In conclusion, we agree with the authors that carefully selected patients with HCM who have significant obstructive pathology and symptoms that are refractory to medical therapy appear to benefit from alcohol septal ablation, and that such therapy is both safe and effective in reducing gradients and progression of symptoms. Importantly, this benefit seems to extend to the cohort of patients with seemingly more mild (NYHA Class II) symptoms, and as a result, we advocate for comprehensive evaluation of patients with hypertrophic obstructive cardiomyopathy using both subjective and objective measurements to determine the extent of symptoms and impact on quality of life, before making decisions about septal reduction therapies. Further study is needed to determine which of these tools are most effective in determining who might benefit from septal reduction therapies.

## Disclosures

None.
